# Acceleration of leaf senescence is slowed down in transgenic barley plants deficient in the DNA/RNA-binding protein WHIRLY1

**DOI:** 10.1093/jxb/erw501

**Published:** 2017-01-28

**Authors:** Weronika Kucharewicz, Assaf Distelfeld, Wolfgang Bilger, Maren Müller, Sergi Munné-Bosch, Götz Hensel, Karin Krupinska

**Affiliations:** 1 Institute of Botany, Christian-Albrechts-University of Kiel, Kiel, Germany; 2 Department of Molecular Biology and Ecology of Plants, University of Tel Aviv, Tel Aviv, Israel; 3 Department of Evolutionary Biology, Ecology and Environmental Sciences, University of Barcelona, Barcelona, Spain; 4 Plant Reproductive Biology, Leibniz Institute of Plant Genetics and Crop Plant Research (IPK), Seeland/OT Gatersleben, Germany

**Keywords:** Barley, chloroplast proteins, DNA/RNA-binding protein WHIRLY1, high light, leaf senescence, stay-green.

## Abstract

WHIRLY1 in barley was isolated as a potential regulator of the senescence-associated gene *HvS40*. In order to investigate whether the plastid–nucleus-located DNA/RNA-binding protein WHIRLY1 plays a role in regulation of leaf senescence, primary foliage leaves from transgenic barley plants with an RNAi-mediated knockdown of the *WHIRLY1* gene were characterized by typical senescence parameters, namely pigment contents, function and composition of the photosynthetic apparatus, as well as expression of selected genes known to be either down- or up-regulated during leaf senescence. When the plants were grown at low light intensity, senescence progression was similar between wild-type and RNAi-W1 plants. Likewise, dark-induced senescence of detached leaves was not affected by reduction of WHIRLY1. When plants were grown at high light intensity, however, senescence was induced prematurely in wild-type plants but was delayed in RNAi-W1 plants. This result suggests that WHIRLY1 plays a role in light sensing and/or stress communication between chloroplasts and the nucleus.

## Highlight

Transgenic barley plants with an RNAi-mediated knockdown of the gene encoding the plastid–nucleus-located DNA/RNA-binding protein WHIRLY1 display a stay-green phenotype at high light intensity, but not at low light.

## Introduction

Senescence is the final phase of development leading to the death of the tissues, organs, and whole plants. Leaf senescence under optimal growth conditions is a part of the normal organ ontogenesis, although it can be prematurely induced by abiotic and biotic stresses. Internal factors controlling the senescence syndrome include age, changes in the levels of plant hormones, and reproductive growth (Buchanan-Wollaston *et al.*, 2005; Guo and Gan, 2012). The complex process is characterized by an organized disassembly of chloroplasts and remobilization of the nitrogen released from degraded chloroplast proteins to younger plant parts (Hörtensteiner and Feller, 2002; Masclaux-Daubresse *et al.*, 2008). Yellowing of the leaf resulting from chlorophyll catabolism and loss of photosynthetic capacity are the first symptoms of senescence, preceding alterations in gene expression and protein amounts (Humbeck *et al.*, 1996). Chloroplasts are the site of photosynthesis and of a variety of reactions essential for plant growth. Information on the operation of these activities is transmitted to the nucleus to adjust gene expression during different stages of development and in the context of environmental cues (Pfannschmidt and Munné-Bosch, 2013; Chan *et al.*, 2016). Genetic reprogramming of senescing leaves leads to a down-regulation of a large number of genes that are expressed in non-senescent leaves, in particular photosynthesis-related genes, and to an up-regulation of a large subset of genes termed senescence-associated genes (SAGs) (Buchanan-Wollaston *et al.*, 2005).

To obtain insight into the regulation of senescence, up-regulated genes encoding transcription factors are of particular interest. In Arabidopsis, the majority of these transcription factors belong to the families of NAC and WRKY factors (Balazadeh *et al.*, 2008). A set of *NAC* genes implicated in regulation of leaf senescence in barley have been identified (Christiansen *et al.*, 2011), whereas from the WRKY family, *HvWRKY12* was found among the transcription factors with the highest up-regulation during barley flag leaf senescence (Hollmann *et al.*, 2014). Another barley gene encoding a potential regulator of leaf senescence is *HvS40*, which is highly expressed under different situations of senescence ranging from dark-induced senescence of detached leaves to age-dependent senescence of flag leaves from field-grown barley plants (Becker and Apel, 1993; Humbeck *et al.*, 1996; Kleber-Janke and Krupinska, 1997; Krupinska *et al.*, 2002), as well as under abiotic and biotic stress conditions (Shi *et al.*, 2012) or in response to hormones (Krupinska *et al.*, 2002, 2014*a*). Senescence-associated expression of *HvS40* is accompanied by specific histone modifications at its promoter (Ay *et al.*, 2015). In fusion with the *uidA* gene encoding β-glucuronidase (GUS), the HvS40 protein was located in the nucleus (Krupinska *et al.*, 2002). A T-DNA insertion in the promoter of the homologous *AtS40-3* gene caused a stay-green phenotype in *Arabidopsis thaliana*, supporting the idea that the S40 proteins are regulators of senescence (Fischer-Kilbienski *et al.*, 2010).

Two striking motifs consisting of inverted repeat sequences (IR2; TGTCA) and W-box elements ([T]TGAC[C/T]) were identified in the promoter of *HvS40* (Krupinska *et al.*, 2014*a*). In an attempt to identify a WRKY transcription factor regulating senescence-associated expression of the *HvS40* gene, the W-box motif was used for binding of nuclear proteins. By this approach, WHIRLY1 was identified (Krupinska *et al.*, 2014*a*), which had been first reported as a component of PBF-2 (*PR-10a* binding factor 2) that upon pathogen attack and after salicylic acid treatment activates the pathogenesis-related *PR10a* gene in potato (Desveaux *et al.*, 2000). PBF-2 is a homotetramer of WHIRLY1 protomers, which was shown to bind to a sequence named elicitor response element (ERE) including W-box elements (Desveaux *et al.*, 2000). Further studies demonstrated a requirement for the 3' ERE half (GTCAAAAA/T, the PBF-2 binding core motif) for the functionality of the element. This motif is found in promoters of several Arabidopsis defense genes induced by salicylic acid (Desveaux *et al.*, 2004). Salicylic acid is known to accumulate in senescing leaves and to induce expression of a subset of SAGs in Arabidopsis (Morris *et al.*, 2000) as well as the barley *HvS40* gene (Krupinska *et al.*, 2002). Regulation of SAGs by salicylic acid indicates an overlap between senescence and stress responses (Guo and Gan, 2012).

Although WHIRLY1 was first characterized as a nuclear transcription factor involved in salicylic acid-dependent expression of *PR* genes (Desveaux *et al.*, 2000, 2004), it has also been detected in chloroplasts (Grabowski *et al.*, 2008). Studies with transplastomic tobacco plants synthesizing a tagged version of the protein inside chloroplasts suggested that WHIRLY1 is translocated from chloroplasts to the nucleus (Isemer *et al.*, 2012). In chloroplasts of Arabidopsis and barley, WHIRLY1 was shown to be a part of transcriptionally active chromosomes (Pfalz *et al.*, 2006; Melonek *et al.*, 2010). Furthermore, WHIRLY1 was shown to be one of most abundant proteins of plastid nucleoids in both maize and Arabidopsis (Majeran *et al.*, 2012; Huang *et al.*, 2013). It was shown to bind non-specifically to plastid DNA and specifically to a set of RNAs deriving from intron-containing plastid genes in maize and barley (Prikryl *et al.*, 2008; Melonek *et al.*, 2010). In Arabidopsis, WHIRLY1 is required for the stability of the plastid genome (Maréchal *et al.*, 2009), and in barley it was found to compact nucleoids and to regulate plastid DNA copy number (Krupinska *et al.*, 2014*b*). Dual localization of WHIRLY1 makes it an interesting candidate for transducing signals from chloroplasts to the nucleus (Foyer *et al.*, 2014) during normal leaf development and in various stress situations.

In this study, the influence of WHIRLY1 on leaf senescence has been investigated using transgenic barley plants with an RNAi-mediated knockdown of the gene (Melonek *et al.*, 2010; Krupinska *et al.*, 2014*b*). Ample physiological and molecular evidence indicates that WHIRLY1 does not affect age- and dark-dependent senescence, but rather is involved in premature induction of senescence processes in response to photooxidative stress.

## Materials and methods

### Plant material and growth conditions

Grains of *Hordeum vulgare* L., cv. Golden Promise (wild type) and *WHIRLY1* RNAi knockdown plants (lines W1-1, W1-7, and W1-9; Krupinska *et al.*, 2014*b*) were imbibed in the dark at 6 °C for 2 d on wet Whatman paper (Carl Roth, Karlsruhe, Germany) followed by germination in the dark at room temperature for 1 d. Barley seedlings were then transferred onto soil in 5 liter pots (8–9 seedlings per pot) (Einheitserde ED73, Einheitswerk Werner Tantau, Uetersen, Germany) and grown in a climate chamber with a 16/8 h light/dark regime (21 °C/18 °C) and ~65% air humidity. For investigations on natural senescence, plants were grown either at low or at high irradiance, ranging from 90 μmol photons m^–2^ s^–1^ to 150 μmol photons m^–2^ s^–1^ for the low light condition and from 310 μmol photons m^–2^ s^–1^ to 390 μmol photons m^–2^ s^–1^ (~350 μmol photons m^–2^ s^–1^ on average) for the high light condition, respectively. The irradiance was measured horizontally. Preliminary experiments were conducted on plants grown at low light till 14 days after sowing (das) and transferred to high light afterwards. To minimize the influence of the different irradiances across the tables, the pots with the plants were randomly rotated in the climate chamber and watered as required. For induction of senescence by darkness, leaves were detached from plants with a razor blade 14 das, put into beakers with water, and incubated in darkness for up to 4 days in the climate chamber with humidity and temperature conditions as described above. Primary foliage leaves used for RNA, protein, and pigment extraction were always collected at midday, immediately frozen in liquid nitrogen, and, after storage at −80 °C, ground in liquid nitrogen.

### SPAD measurement

Changes in the chlorophyll content of leaves were monitored non-invasively using the Chlorophyll-meter SPAD-502 plus (Konica Minolta, Tokyo, Japan). Each leaf was measured at three positions: at the tip, the middle, and close to the leaf base. Mean values were calculated from 15–25 leaves per genotype (wild-type and RNAi-W1 plants).

### Chlorophyll fluorescence measurements

Maximum efficiency of PSII photochemistry (*F*_v_/*F*_m_) was determined by fluorescence measurements using a pulse amplitude modulated fluorometer (IMAGING-PAM Chlorophyll Fluorometer; Heinz Walz GmbH, Effeltrich, Germany, http://www.walz.com) according to the saturation pulse method described by Schreiber *et al.* (1986) and Genty *et al.* (1989). Before measuring *F*_v_/*F*_m_, 3–5 leaves were detached, covered with wet paper, and kept for at least 15 min in the dark. The experiment was done three times.

### Gas exchange measurements

The CO_2_ assimilation rate [*A* (μmol m^–2^ s^–1^)] was measured using a portable Gas Exchange Fluorescence System GFS-3000 (Heinz Walz GmbH) at ambient light conditions in the climate chamber ~1 h after the lights were turned on in the morning. Settings of the instrument were as follows: flow rate 750 μmol min^–1^, impeller 7, CO_2_ 380 ppm, 21 °C cuvette temperature, relative humidity 65%. All calculations were done according to the equations given in the manual (Walz GmbH 2013).

### Determination of pigment contents

Leaf segments were cut from the middle area of the leaf (2–3 cm from the leaf tip) and stored in a freezer at −80 °C until analysis by HPLC. For extraction, the leaf segments were ground in the frozen state along with three metal beads in a Geno Grinder (Type 2000; SPEX CertiPrep, Munich, Germany) with 0.9 ml of 80% (v/v) acetone buffered with 20 mM Tris, pH 7.8. After centrifugation, the pellet was extracted twice with 300 μl of pure acetone. From the unified extracts, 50 μl were used for HPLC analysis on an Agilent 1100 system (Agilent, Waldbronn, Germany) with detection with a Diode Array Detector (DAD). The protocol was used as described by Niinemets *et al.* (1998).

### Analyses of promoter sequence elements

Promoters of *HvS40*, barley *WRKY12*, *PR1*, and *PR10* genes were screened for the presence of EREs known to be bound by WHIRLY1 (Despres *et al.*, 1995) and W-box elements employing the Geneious v 8.0.5 software. Additionally a New PLACE database was used to search for I-box elements associated with light-responsive promoter regions (Higo *et al.*, 1999). The 2 kb promoter regions were acquired either from the NCBI (National Center for Biotechnology Information) or from ENA (European Nucleotide Archive) database and extracted from sequences with the following accession numbers: FI496090 and AC264417.1 for *S40*, morex_contig_2547137 for *WRKY12*, bowman_contig_114183 for *PR1*, and morex_contig_145303 for *PR10*.

### RNA extraction and expression analysis

Total RNA was extracted using either the TRIzol-based method as described by Ay *et al.* (2008) or the Spectrum™ Plant Total RNA Kit (Sigma Aldrich, St. Louis, MO, USA) according to the manufacturer’s instructions. To remove contamination by genomic DNA, the isolated RNA was treated with DNase I (MBI Fermentas, St. Leon-Rot, Germany) or an On-Column DNase I Digestion Set (Sigma Aldrich). The concentration was measured by a Nanodrop 200 instrument (Thermo Scientific, Peqlab, Erlangen, Germany). The quality of the RNA was examined by separation on a 1.5% (w/v) agarose gel and staining by ethidium bromide. A 100 ng aliquot of total RNA from each sample was subjected to gene expression analysis using the Nanostring system (Nanostring Technologies, Seattle, WA, USA). The ‘Nanostring Technology’ uses fluorescent colour-coded ‘molecular barcode’ oligonucleotides that hybridize directly to the target molecules as described in detail by Geiss *et al.* (2008). The CodeSet was designed to consist of three reference genes and 27 genes of interest (see Supplementary Table S1 at *JXB* online). Probe sets for each gene in the CodeSet were designed and synthesized at Nanostring Technologies. The three reference genes were *CDC*, *HvACTIN*, and *RLI* (Giménez *et al.*, 2011). The experimental genes included transcription factor-, photosynthesis-, stress-, remobilization-, and degradation-related genes (Table 1). The probes were complementary to a 100 base region of the target mRNAs. Sequences for probes used in nCounter analysis are listed in Supplementary Table S1. All procedures related to mRNA quantification, including sample preparation, hybridization, detection, scanning, and data calculation, were carried out as recommended by Nanostring Technologies. Raw Nanostring counts for each gene were subjected to a technical normalization using the counts obtained for six positive control probe sets. The background to be subtracted was calculated based on eight negative control probe signals prior to a biological normalization using the three housekeeping genes included in the CodeSet. Normalized data were log2-transformed and visualized as a heatmap using MeV 4.9 (http://mev.tm4.org/)

**Table 1.** Genes selected for expression analyses, accession numbers, and brief description of their functions

Category	Gene name	Accession number	Description
*WHIRLY* genes and predicted WHIRLY1 target genes	*Whirly1*	AK365452.1	DNA repair, regulation of transcription, defense response, telomere maintenance
	*Whirly2*	AK249976.1	DNA repair, regulation of transcription, defense response
	*S40*	AK248955.1	Senescence-associated gene
	*WRKY12*	AK354853.1	WRKY family transcription factor
	*PR1*	Z21494.1	Pathogenesis-related gene 1
	*PR10*	AY220734.1	Pathogenesis-related gene 10
Photosynthesis	*RbcS*	U43493.1	Rubisco small subunit
	*LHCA1*	AF218305.1	PSI type I Chl *a*/*b*-binding protein
	*PsaD*	M98254.1	PSI-D subunit of PSI
	*LHCB4*	AJ006296.1	CP29 Chl *a*/*b*-binding protein of plant PSII
	*ELIP58*	X15693.1	Early light inducible protein
Transcription factors	*NAC005*	AK251058.1	NAC family transcription factor
	*NAC029*	EU908210.1	NAC family transcription factor
	*MYB*	AK367373.1	MYB-related transcription factor family
ROS metabolism	*APX1*	AJ006358.1	Ascorbate peroxidase
	*CAT1*	U20777.1	Catalase2
	*CAT2*	U20778.1	Catalase3
	*Cu-ZnSOD1*	HM537232.1	Superoxide dismutase, chloroplastic
	*FeSOD1*	AK375983.1	Superoxide dismutase, chloroplastic
	*ptOX*	AK359478.1	Alternative oxidase, chloroplastic
ABA metabolism	*ABA8OH-1*	DQ145932.1	ABA degradation, ABA 8'-hydroxylase
	*NCED2*	DQ145931.1	ABA biosynthesis, 9-*cis*-epoxycarotenoid dioxygenase
Remobilization and degradation	*ATG8*	AK251678.1	Autophagy gene 8
	*GS1*	KF815945.1	Glutamine synthetase 1, cytosolic
	*GS2*	AK360336.1	Glutamine synthetase 2, plastidial
	*SAG12*	AM941123.1	Senescence-associated gene 12, cysteine protease
	*PAP14*	AM941124.1	Papain-type cysteine peptidase
	*PAO*	AK365037.1	Pheophorbide *a* oxygenase, chlorophyll degradation
Reference genes	*Act*	AK248710.1	Actin
	*CDC*	AK376851	Cell division control protein
	*RLI*	AK355298	RNase L inhibitor-like protein

For the qRT-PCR analysis, cDNA was prepared from equal amounts of the total RNA using a QuantiTect Reverse Transcription Kit (Qiagen, Hilden, Germany) as described by the manufacturer. Quantitative PCR was performed in a volume of 12 µl using a Rotor-Gene SYBR Green PCR Kit (Qiagen) in a Rotor-Gene Q cycler (Qiagen). Primers used for the qPCR analysis are detailed in Supplementary Table S2. The thermocycling conditions consisted of 5 min incubation at 95 °C, followed by 40 two-temperature cycles of 5 s at 95 °C and 10 s at 60 °C, and a temperature gradient from 50 °C to 99 °C for the melting curve. Three biological samples each were analysed with three technical replicates per sample. The relative expression changes for *ELIP58* (normalized to *HvSP2* and *HvActin* as reference genes and to the wild type at the first time point as a control sample) were determined using the Rotor-Gene Q Series Software (Qiagen) and the ΔΔCt method (Livak and Schmittgen, 2001).

### Immunological analyses of protein abundances

Proteins were extracted from 500 mg of whole primary foliage leaves with a double volume (1 ml) of sample buffer [62.5 mM Tris–HCl, pH 6.8, 10% (v/v) glycerol, 1% (w/v) SDS, 5% (v/v) ß-mercaptoethanol]. After heating the suspensions at 65 °C for 5 min, cellular debris was removed by centrifugation. Equal amounts of protein (10 µg) were subjected to SDS–PAGE, and separated proteins were transferred to nitrocellulose membranes (Carl Roth, Roti-NC) by electroblotting (Humbeck *et al.*, 1994). After incubation with the primary antibody, immunoreactive bands were visualized using a peroxidase-coupled secondary antiserum with chemiluminescence detection (ECL Select Amersham, Pierce Thermo Scientific Waltham, MA, USA; Ultra TMA-6 Lumigen, Southfield, MI, USA). Primary antibodies were directed towards HvWHIRLY1 (Grabowski *et al.*, 2008, 2nd peptide), LHCB1 (Agrisera AS01004), LHCB4 (Humbeck and Krupinska, 2003), D1 (Agrisera AS01016), LHCA1 (Agrisera AS01005), FNR (AntiProt F01A-1), Cu/ZnSOD (Agrisera AS06170), and an antibody directed towards PSI (kindly provided by Poul Eric Jensen, University of Copenhagen, Denmark).

## Results

### Leaf development and senescence in *WHIRLY1* knockdown plants

Barley WHIRLY1 has been detected as a protein binding *in vitro* to an ERE-like element, which is present in the promoter of the barley senescence marker gene *HvS40* (Krupinska *et al.*, 2014*a*). To investigate whether barley plants with a reduced amount of WHIRLY1 (Krupinska *et al.*, 2014*b*) differ from wild-type plants with regard to senescence, three independent transgenic lines with an RNAi-mediated knockdown of the gene, RNAi-W1-1, RNAi-W1-7, and RNAi-W1-9, were compared with the wild type. When the plants were grown in a glasshouse during summer, senescence was delayed in plants of all three RNAi-W1 lines (Supplementary Fig. S1).

To investigate further the impact of WHIRLY1 on senescence, primary foliage leaves of seedlings of the wild type and of the RNAi-W1-7 line showing the most severe knockdown (Krupinska *et al.*, 2014*b*) were characterized during growth in a climate chamber with controlled temperature and light conditions. Senescence of the primary foliage leaves was described by their relative chlorophyll contents measured by SPAD, contents of pigments and their photosynthetic activity determined by chlorophyll fluorescence measurements (*F*_v_/*F*_m_) and CO_2_ assimilation rate (*A*).

**Fig. 1.**
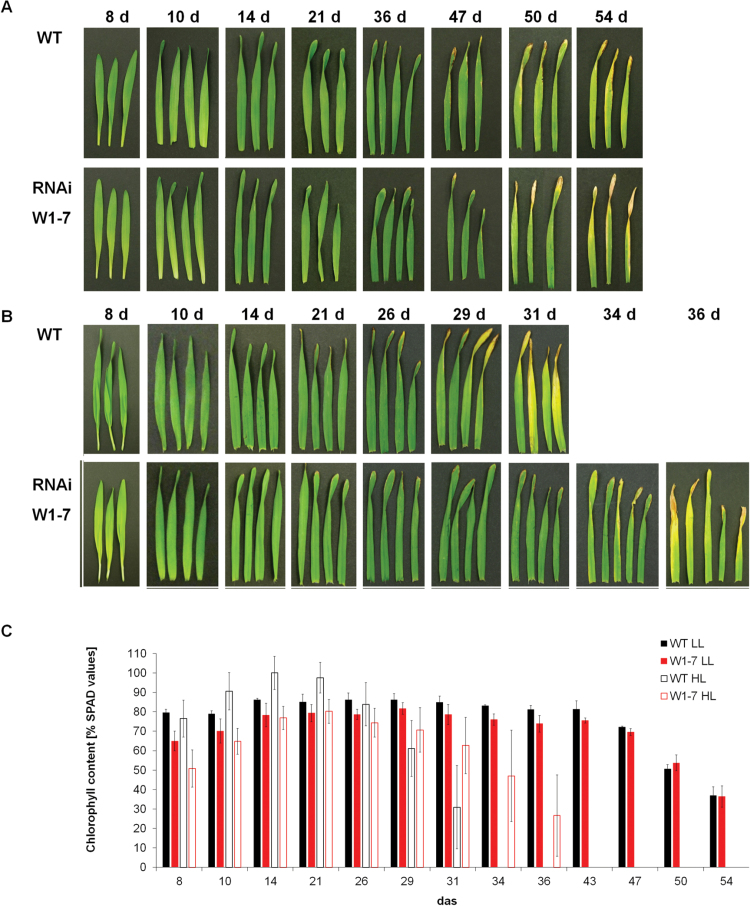
Irradiance-dependent impact of the RNAi-mediated knockdown of *WHIRLY1* on development and senescence of primary foliage leaves. Leaves were cut from plants of the wild type (WT) and the RNAi-WHIRLY1 line 7 (W1-7) grown either under low- (LL) or high-light conditions (HL) (120 μmol m^–2^ s^–1^ and 350 μmol m^–2^ s^–1^, respectively). Primary foliage leaves were characterized by photography (A, B) and by their relative chlorophyll levels (SPAD values) (C). When plants were grown at low light, characterization was done from 8 to 54 days after sowing (das). When plants were grown at high light, characterization was done from 8 to 36 das. Columns represent SPAD values measured on leaves grown at low light (black, wild type; red, RNAi-W1-7) or at high light (open bars with black lines: wild type, openbars with red lines: RNAi-W1-7. Each measurement was done on 15–25 leaves in three independent experiments.

**Fig. 2.**
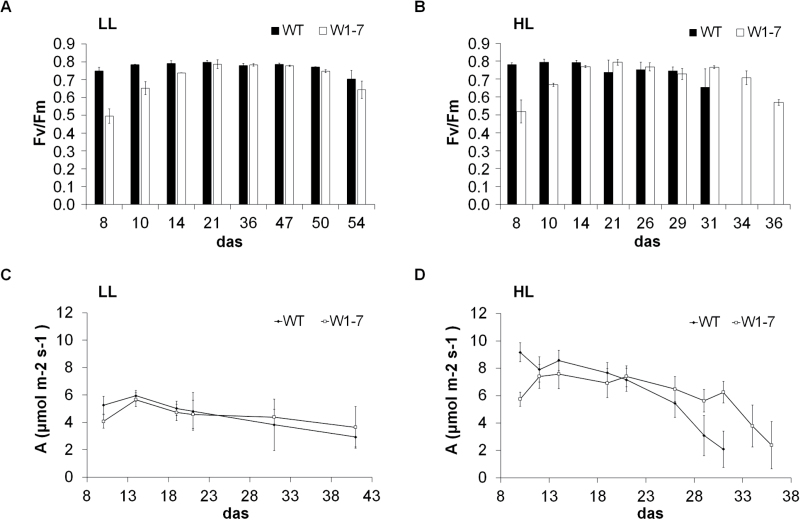
Developmental changes in PSII efficiency measured as *F*_v_/*F*_m_ (A, B) and in photosynthetic CO_2_ uptake (C, D). Measurements were performed with leaves collected from wild-type and RNAi-W1-7 plants grown either at low (LL, 120 μmol s^–1^ m^–2^, A, C) or high (HL, 350 μmol s^–1^ m^–2^, B, D) irradiance. PSII efficiency was determined on 9–15 excised leaves; photosynthetic assimilation was measured *in situ* on 15 attached leaves of each genotype.

When the seedlings were grown at low irradiance (120 μmol s^–1^ m^–2^), no apparent phenotypic differences were detectable between leaves of the wild type and the RNAi-W1-7 line (Fig. 1A). SPAD values started to decrease at 43 das in both genotypes (Fig. 1C), followed by a slight decrease of *F*_v_/*F*_m_ at 50 das (Fig. 2A). From 8 until 43 das, the chlorophyll content of primary foliage leaves of RNAi-W1-7 plants was slightly lower in comparison with those of the wild type. Until 14 das, this difference was accompanied by corresponding differences in *F*_v_/*F*_m_ although from 21 das PSII efficiency was similar between RNAi-W1-7 and wild-type leaves. The CO_2_ assimilation rates were also comparable between the genotypes (Fig. 2C).

When the plants were grown at high irradiance (350 μmol s^–1^ m^–2^), wild-type leaves had a significantly shorter lifetime than leaves of the RNAi-W1-7 line (Fig. 1B). SPAD values of leaves of the wild type and the RNAi-W1-7 line started to decline at 21 and 26 das, respectively (Fig. 1C). In primary foliage leaves of the RNAi-W1-7 plants, the relative chlorophyll content barely differed between low-light-grown plants and high-light-grown plants between 10 and 21 das. In contrast, the relative chlorophyll content of the high-light-grown wild type exceeded the values measured for leaves of low-light-grown plants in the same period (Fig. 1C). The differences in chlorophyll content between wild-type and transgenic plants under high light were accompanied by differences in photosynthesis as determined by gas exchange and PSII efficiency measurements. Leaves of RNAi-W1-7 plants had lower CO_2_ assimilation rates until 21 das, whereas from 29 to 36 das they assimilated higher CO_2_ amounts than the wild type, whose leaves were already senescent (Fig. 2D). For both genotypes, the assimilation rates positively correlated with the values for chlorophyll contents and *F*_v_/*F*_m_, indicating that lower assimilation rates were caused by both loss of chlorophyll and a reduced PSII activity.

### Pigment composition

**Fig. 3.**
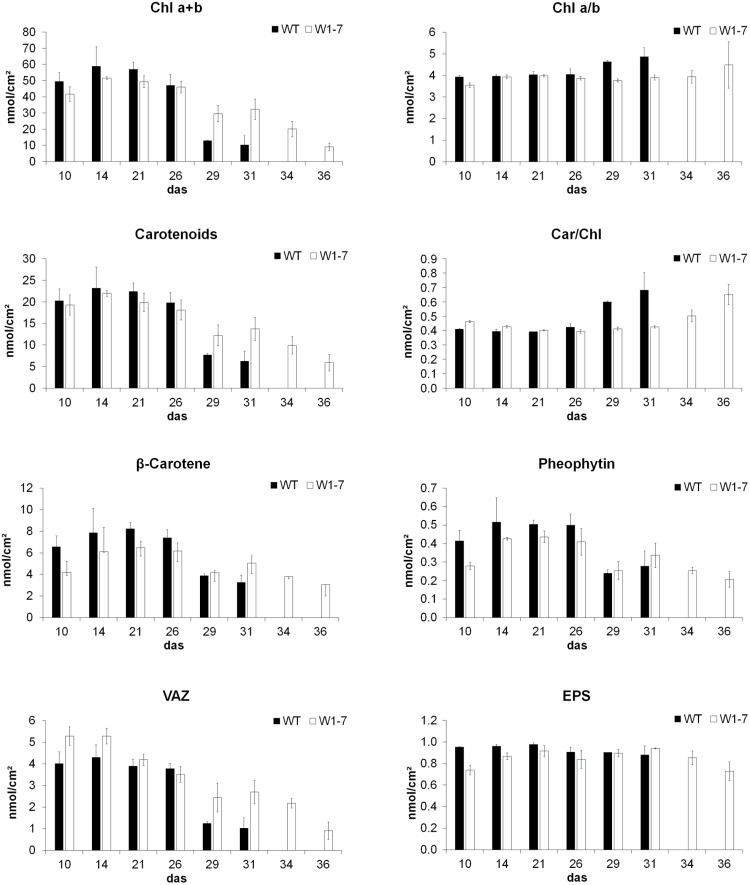
Developmental changes in pigment composition of primary foliage leaves from wild-type and RNAi-W1-7 plants. Plants were grown at high light (350 μmol m^–2^ s^–1^). Pigment analyses by HPLC were done during the period from 10 to 36 das with extracts from segments cut from the middle of the leaves. Besides total chlorophyll content (Chl *a*+*b*) and the total contents of carotenoids, the ratios of Chl *a* to *b* as well as the ratios of carotenoids to chlorophylls (Car/Chl) were calculated. In addition the values for ß-carotene, pheophytin, the VAZ pool (violaxanthin, antheraxanthin, zeaxanthin), and its epoxidation state (EPS) are depicted. Pigment contents were measured on three biological replicates.

Analysis revealed that the chlorophyll content of leaves from RNAi-W1-7 plants did not reach the high levels observed for wild-type leaves at 14 and 21 das (Fig. 3). Chl *a*/*b* ratios barely changed during senescence in both genotypes, although they were lower in young leaves of the RNAi-W1 plants in comparison with wild-type plants at 10 das (Fig. 3). Total carotenoid content was slightly lower in W1-7 leaves until 29 das, when the carotenoid levels strongly declined in the wild type (Fig. 3). The ratio of carotenoids to chlorophylls at 10 das was higher in W1-7 leaves than in wild-type leaves, and this ratio increased in senescent leaves of both genotypes (Fig. 3). The content of ß-carotene as well as of pheophytin was reduced in the transgenic leaves compared with the wild type (Fig. 3). The VAZ (violaxanthin, antheraxanthin, zeaxanthin) pool was elevated in both young (10 and 14 das) and senescent (29 and 31 das) leaves of transgenic plants (Fig. 3G). The epoxidation state (EPS) was lower in young leaves of RNAi-W1-7 plants than in wild-type leaves, but at later stages no differences were detectable between the two genotypes (Fig. 3).

### Promoter analysis of potential WHIRLY1 target genes and gene expression analyses

**Fig. 4.**
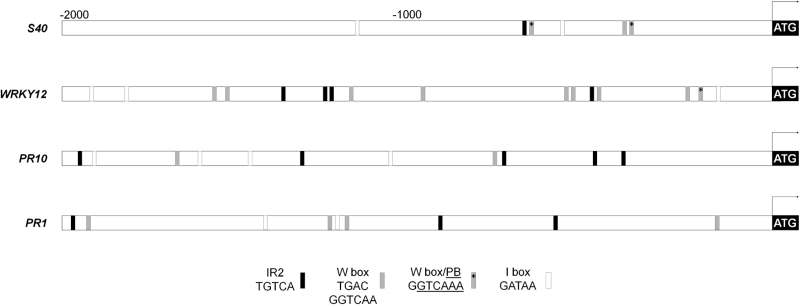
Schematic representation of sequence elements in the promoters of the barley genes *S40*, *WRKY12*, *PR10*, and *PR1*. Black boxes represent the inverted repeat IR2 sequence of the elicitor response element (ERE), and grey boxes mark W-box elements (TGAC core as well as GGTCAA); grey boxes with asterisks mark W-boxes overlapping with the PB element; and white boxes represent I-box elements typical for light-regulated promoters.

Originally, WHIRLY1 was identified as a transcriptional activator of the *PR10a* gene of potato (Desveaux *et al.*, 2000). In barley WHIRLY1 had been described as a protein binding to the promoter of the *HvS40* gene (Krupinska *et al.*, 2014*a*). The sequence proposed to be required for binding of WHIRLY1 includes an inverted repeat (IR2, TGTCA; Krupinska *et al.*, 2014*a*) element of the ERE (IR1-IR2: TGACAnnnnTGTCA, Desveaux *et al.*, 2000) overlapping with a W-box [(C/T)TGAC(C/T), Eulgem *et al.*, 2000] and the PB motif (PBF-2 binding element, GTCAAAAA/T, Desveaux *et al.*, 2004). Association of WHIRLY1 with the ERE-like element might indicate that the expression of *HvS40* is regulated by WHIRLY1 (Krupinska *et al.*, 2014*a*). As expected, ERE sequences have also been detected in the promoters of the barley *PR10a* and *PR1* genes (Fig. 4). Furthermore, the promoter of barley *WRKY12* showing the highest expression among the *WRKY* genes during barley leaf senescence (Hollmann *et al.*, 2014) was found to possess ERE-like elements similar to those in the promoter of *HvS40* (Fig. 4), thus suggesting that *WRKY12* might be a potential target of WHIRLY1. Combinations of ERE and W-boxes in near proximity to each other were found only in the promoters of *HvS40*, *HvWRKY12*, and *HvPR10a*, whereas PB-like motifs occurred in the promoters of *HvS40* and *HvWRKY12* (Fig. 4). Analyses with the New PLACE database revealed that all four promoters contain 2–4 I-box elements. Such boxes have been proposed to be crucial for light-induced expression of many photosynthesis-associated nuclear genes (PhANGs), such as *RBCS* and *LHCB* (López-Ochoa *et al.*, 2007).

To investigate whether the differences in leaf senescence between wild-type and RNAi-W1-7 plants under high light are accompanied by changes in expression of the putative WHIRLY1 target genes, mRNA levels of *HvS40*, *HvWRKY12*, *HvPR10*, and *HvPR1* were determined at different times after sowing using Nanostring technology, which allows for a very precise quantification of mRNA molecules (Geiss *et al.*, 2008). Besides the predicted WHIRLY1 targets, expression of *WHIRLY1* and *WHIRLY2* as well as genes belonging to five functional categories expected to be important in senescence was examined (Table 1). In the case of the wild type, leaf samples were taken at 10, 14, 21, 26, 29, and 31 das. In addition to these time points, samples from RNAi-W1-7 plants were also taken at 34 and 36 das. With regard to the chlorophyll content, the last sample (36 das) collected from the RNAi-W1-7 plants corresponded to the last sample taken from the wild-type plants (31 das) grown under high light (Fig. 3).

**Fig. 5.**
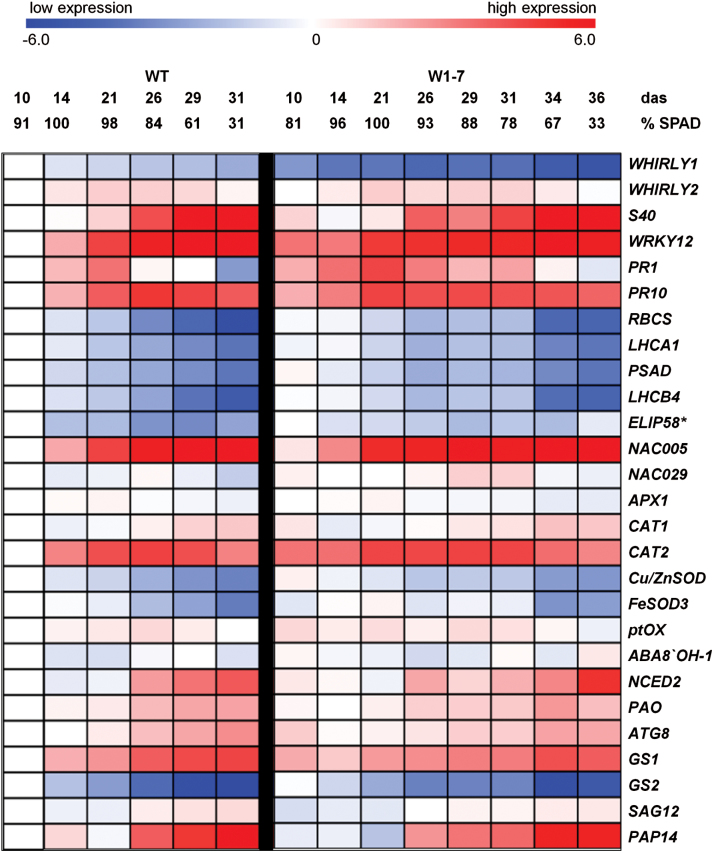
Expression of selected genes in primary foliage leaves of wild-type and RNAi-W1-7 plants grown at high irradiance (350 μmol m^–2^ s^–1^). Expression of 26 genes except *ELIP58* was analysed by Nanostring technology. Expression of *ELIP58* (*) was determined by quantitative PCR. The analysis was done on three biological replicates. RNA was extracted either from leaves of wild-type plants grown for 10, 14, 21, 26, 29, and 31 das or from leaves of RNAi-W1-7 plants grown for the same times and in addition for 34 and 36 das. The relative chlorophyll content of the leaves is indicated by SPAD values. The expression ratios (each sample was compared with the wild-type sample at 10 das as reference) were log2-transformed and visualized as a heatmap using MeV 4.9, whereby –6 (dark blue) is strongly decreased expression, 0 means no change, and 6 (red) represents a highly increased transcript level.

Taking into consideration that the RNAi-W1-7 plants were not able to accelerate senescence in response to high light, WHIRLY1 might play a role in retrograde signalling which during senescence as well as during chloroplast development was proposed to adjust nuclear gene expression to the functional state of chloroplasts (Pfannschmidt and Munné-Bosch, 2013). Therefore, nuclear-encoded photosynthesis-associated genes known to be controlled by retrograde signals were included in the set of genes, namely *RBCS*, *LHCB4*, *LHCA1*, *PSAD*, and *ELIP58* (Pfannschmidt *et al.*, 2001; Gray *et al.*, 2003; Osipenkova *et al.*, 2010). *RBCS* encodes the small subunit of Rubisco, and LHCB4, LHCA1, and PSAD are components of the photosystems. ELIP58 is a putative chlorophyll chaperone and its gene is known to be expressed during exposure to high light (Rus Alvarez-Canterbury *et al.*, 2014). In addition, the set included genes reported to play roles in regulation and execution of leaf senescence. Among this group are genes encoding transcription factors reported to be up-regulated during barley leaf senescence, namely *NAC005*, *NAC029* (*NAM-B1*, putative orthologue of *AtNAP*), and *MYB* (Christiansen and Gregersen, 2014; Distelfeld *et al.*, 2014; Hollmann *et al.*, 2014). With regard to the role of reactive oxygen species (ROS) in regulation of senescence (Zimmermann and Zentgraf 2005; Juvany *et al.*, 2013; Krieger-Liszkay *et al*., 2015), genes encoding enzymes involved in ROS management such as catalases (*CAT1*, *CAT2*), superoxide dismutases (*Cu-ZnSOD1*, *FeSOD1*), ascorbate peroxidase (*APX1*), and plastid terminal oxidase (*ptOX*) were selected. Senescence is known to be controlled by hormones. In particular, abscisic acid (ABA) was shown to accumulate during high light exposure (Galvez-Valdivieso *et al.*, 2009) and to accelerate senescence processes (Gepstein and Thimann, 1980). It is hence likely that the stay-green phenotype of the RNAi-W1 plants is due to a lack of senescence-promoting hormones such as ABA. Therefore, the *NCED2* gene encoding 9-*cis*-epoxycarotenoid dioxygenase, the key enzyme of ABA biosynthesis, and the *ABA8OH-1* gene encoding ABA 8'-hydroxylase involved in ABA degradation (Seiler *et al.*, 2011), were included. Finally, the set contained a group of genes encoding proteins involved in degradation of chloroplast components and remobilization of nitrogen, namely the gene encoding pheophorbide *a* oxygenase (*PAO*), a key enzyme of chlorophyll catabolism (Pruzinska *et al.*, 2005), the two cysteine proteases *SAG12* and *PAP14* (Hollmann *et al.*, 2014), two glutamine synthetase genes (*GS1*, *GS2*), and the autophagy gene *ATG8* (Avila-Ospina *et al.*, 2014, 2015). Detailed Nanostring results are shown on graphs in Supplementary Fig. S2. Overall expression changes for most of the selected genes occurred during an extended time in RNAi-W1-7 samples compared with the wild type (Fig. 5; Supplementary Fig. S2). Nevertheless, minor changes in expression of genes are outlined below.

#### *WHIRLY* genes and predicted *WHIRLY1* target genes

As expected, the level of the *WHIRLY1* transcript was significantly reduced in leaves of the RNAi-W1-7 plants compared with the wild type. The *WHIRLY1* mRNA level was highest in young, still growing wild-type leaves and decreased gradually with leaf maturation and senescence. Expression of *WHIRLY2* was transiently induced in mature and early senescent leaves from both genotypes. *HvS40* and *HvWRKY12* transcript levels increased during leaf senescence in wild-type and transgenic leaves. *HvWRKY12* transcripts accumulated from 14 das, whereas up-regulation of *HvS40* was detected later (26 das). Senescence-associated expression of both genes was rising at later time points in W1-7 leaves in accordance with their delayed senescence phenotype. It is noteworthy that young leaves of *WHIRLY1* knockdown plants at 10 das had higher transcript levels of *HvS40* and *HvWRKY12* in comparison with the wild type. Similarly, mRNA levels of *PR1* and *PR10* were elevated in young leaves (10 das) of RNAi-W1 plants compared with the wild type, with the highest expression in leaves collected between 21 and 26 das. The *PR1* transcripts were more abundant in W1-7 leaves at all stages of leaf development in comparison with the wild type, whereas *PR10* up-regulation was highest in the wild type at 26 das.

#### Photosynthesis

mRNA levels of *RBCS*, *LHCB4*, *LHCA1*, *PSAD*, and *ELIP58* were highest in young leaves of both genotypes, and gradually decreased with time. Transcripts of the photosynthesis-related genes were more abundant in transgenic leaves compared with the wild type at almost all of the time points.

#### Transcription factors

The expression of the gene encoding the transcription factor NAC005 increased during senescence progression in both genotypes starting at 21 das. In comparison, the expression of *NAC029* was low and stable throughout all developmental stages. The expression of *MYB* was almost undetectable, thus it was excluded from the analysis.

#### ROS metabolism

Expression of genes encoding enzymes of the antioxidant system showed similar kinetics in wild-type and RNAi-W1-7 leaves. At 10 das, however, transgenic leaves compared with wild-type leaves had higher levels of *CAT1*, *CAT2*, *Cu-ZnSOD*, and *ptOX* mRNAs. Whereas *CAT1* was up-regulated with progression of senescence, expression of *APX1*, *CAT2* and *ptOX* increased at early senescence and declined at later stages. Expression of both *SOD* genes was highest in young and mature leaves. Thereafter during senescence mRNA levels decreased in both genotypes.

#### ABA metabolism

The level of the *ABA'8OH-1* transcript was largely unaffected during the development of leaves, while expression of *NCED2* increased during senescence starting at 26 das in both the wild type and RNAi-W1-7.

#### Genes involved in degradation and remobilization

*PAO* and *ATG8* transcript levels were up-regulated during senescence in both genotypes. The two genes encoding glutamine synthetase showed inverse expression patterns. While the mRNA level of the cytosolic form (G*S1*) increased with age of the leaves, the mRNA level of the plastid form (*GS2*) declined in both wild-type and RNAi-W1-7 plants. Expression of *ATG8* and *GS1* in young leaves was >2-fold and almost 4-fold higher, respectively, in leaves of the RNAi-W1-7 plants (10 das) compared with wild-type plants. At later stages of development, levels of both transcripts were lower in W1-7 than in wild-type leaves. The mRNA levels of the two protease-encoding genes *SAG12* and *PAP14* increased during senescence in both genotypes. The maximal levels were, however, lower in leaves of RNAi-W1-7 compared with wild-type plants.

### Immunological analyses of photosynthesis-associated proteins

**Fig. 6.**
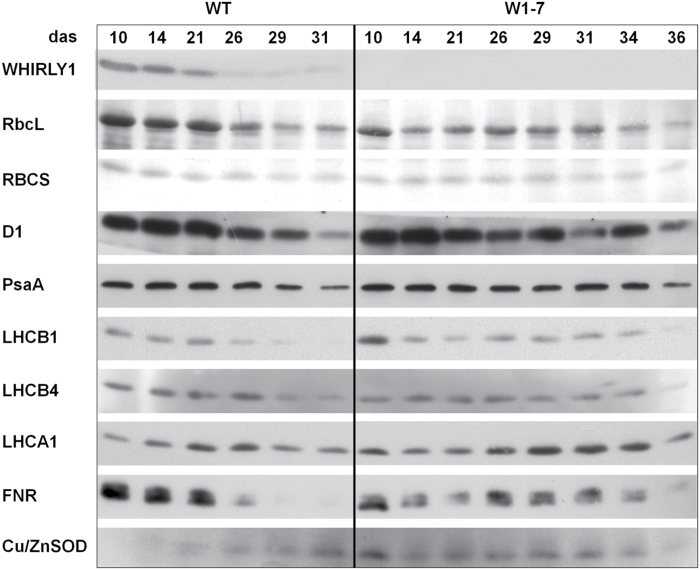
Immunological analyses of the relative amounts of photosynthesis-associated proteins during senescence of primary leaves from wild-type (WT) and RNAi-W1-7 plants grown at high irradiance. Samples were prepared from wild-type plants grown for 10, 14, 21, 26, 29, and 31 das, as well as from RNAi-W1-7 plants grown for 10, 14, 21, 24, 29, 31, 34, and 36 das. The analysis was done with two biological replicates. WHIRLY1, photosynthesis-related proteins, and Cu/ZnSOD were detected by specific antibodies.

A typical feature of leaf senescence is the decline in photosynthesis coinciding with a degradation of the proteins of the photosynthetic apparatus. Senescence-associated changes in protein amounts of RNAi-W1-7 and wild-type plants were analysed by SDS–PAGE and immunoblotting. Immunological analyses were performed with antibodies directed against LHCB1, LHCB4, LHCA1, and reaction centre proteins D1 (PsbA) and PsaA, as well as against ferredoxin oxidoreductase (FNR) catalysing the transfer of electrons from PSI to the terminal acceptor NADP^+^ of the electron transport chain. In addition, the amounts of WHIRLY1 and Cu/ZnSOD were investigated. In a previous study, WHIRLY1 was shown to increase in abundance in parallel with photosynthesis-associated proteins, such as RUBISCO, during chloroplast development (Krupinska *et al.*, 2014*b*). Accordingly, here the amount of WHIRLY1 declined with age of the wild type (Fig. 6).

In the wild type, the decrease in the amount of RUBISCO in senescing leaves progressed more gradually when compared with the rapid decline of WHIRLY1. The decline in the PSII reactions centre protein D1 was as fast as the decline in RUBISCO subunits RbcL and RBCS. In contrast, the reaction centre protein of PSI, PsaA, stayed rather stable. The fastest decline was observed for the FNR proteins. In accordance with previous reports (Humbeck *et al.*, 1996), the amounts of light-harvesting complex apoproteins declined more slowly than RbcL and the D1 protein. In leaves of transgenic plants, the amounts of RUBISCO subunits as well as of the other photosynthesis proteins stayed rather stable until the very last stage of leaf senescence. Immunodetection of chloroplastic Cu/ZnSOD revealed that the amount of the protein increased with senescence in the wild type while it was highly abundant in young leaves of RNAi-W1-7 leaves and declined at later stages of development (Fig. 6).

### Dark-induced senescence

**Fig. 7.**
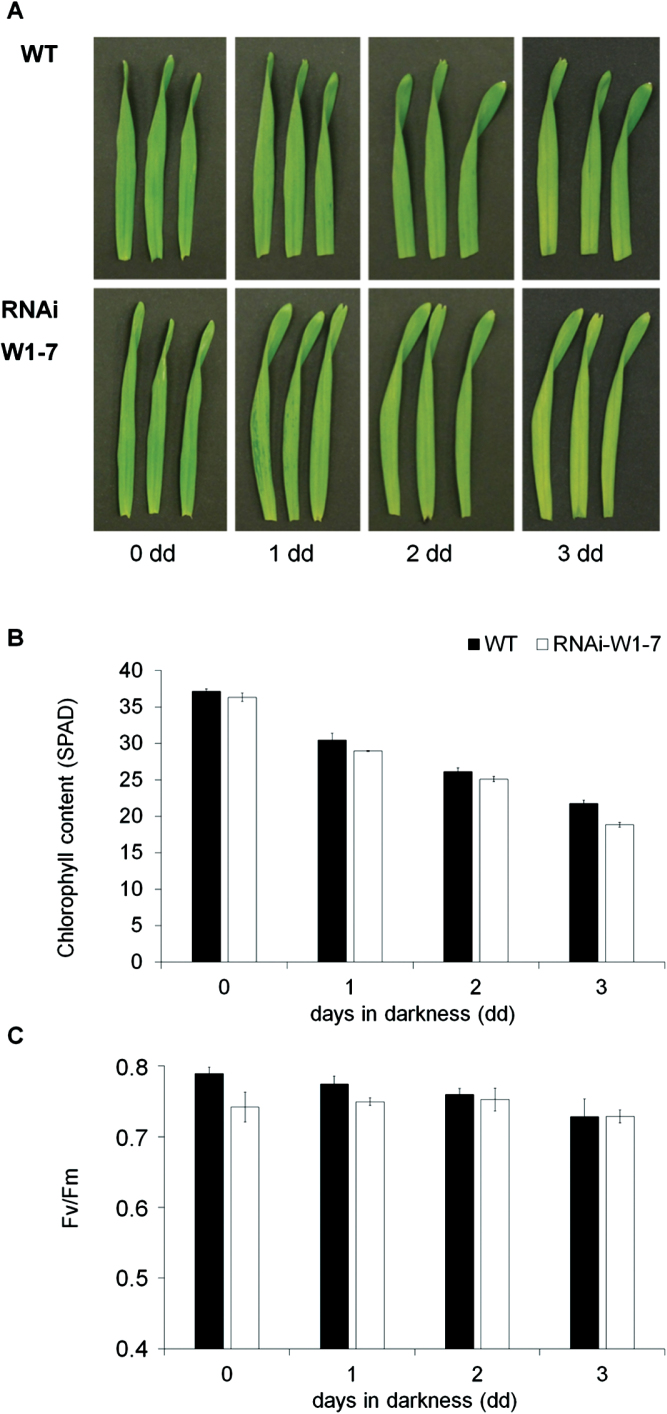
Dark-induced senescence of primary foliage leaves cut from wild-type and RNAi-W1-7 plants. Plants were grown until 14 das at an irradiance of 120 μmol m^–2^ s^–1^. Leaves were cut and transferred to darkness for up to 3 days. (A) Photographic images, (B) relative chlorophyll contents (SPAD), and (C) PSII efficiency measured as *F*_v_/*F*_m_. Mean (±SD values) are based on the values from three independent experiments. Black bars represent wild-type values; white bars are values of the W1-7 RNAi leaves.

Senescence can be induced artificially by transfer of cut leaves to darkness (Becker and Apel, 1993). To investigate whether dark-induced senescence is also retarded in the RNAi-W1-7 plants, detached leaves of 14-day-old plants were transferred to darkness for up to 3 days. Before detachment of leaves, plants were grown in low light (~120 μmol photons m^–2^ s^–1^). Dark treatment led to gradual decreases in chlorophyll content and in PSII efficiency in both wild-type and RNAi-W1-7 leaves, despite the chlorophyll content being slightly lower in leaves of the transgenic line after 3 days of darkness (Fig. 7).

## Discussion

As a plastid–nucleus-located DNA/RNA-binding protein, WHIRLY1 has been suggested to be an ideal candidate for the transfer of information from plastids to the nucleus (Grabowski *et al.*, 2008; Leister, 2012). Although plastid signalling has been mostly studied during chloroplast development, it is likewise important during other periods of development including leaf senescence, and in this context it has been termed ‘degradational control’ (Pfannschmidt and Munné-Bosch, 2013). The results of this study showed that senescence was not altered in transgenic barley plants with an RNAi-mediated knockdown of *WHIRLY1* (RNAi-W1) when the plants are grown at low light or when leaves from the plants were transferred to darkness (Figs 1, 2, 7). When, however, the light intensity was increased, senescence was accelerated in leaves of the wild type more than in leaves of the RNAi-W1 plants which at high light display a ‘stay-green’ phenotype. In a parallel study, the RNAi-W1 plants were shown to display a ‘stay-green’ phenotype during drought-accelerated senescence (Janack *et al.*, 2016).

It is well known that stress conditions and diverse compounds accumulating during stress such as ROS, hormones, and sugars have a promoting impact on senescence processes (Buchanan-Wollaston *et al.*, 2005). Premature senescence in response to stress is a stress avoidance response ensuring that the nutrients in leaves are efficiently remobilized to the seeds. Among the factors promoting senescence are drought and high light. It is likely that they act on senescence via changes in the photosynthetic apparatus and corresponding retrograde signalling (Pfannschmidt and Munné-Bosch, 2013; Dietz, 2015; Chan *et al.*, 2016).

Under high light, the reduced photosynthetic capacity of the RNAi-W1 plants probably leads to an excess excitation energy (EEE) situation (Karpiński *et al.*, 2013; Szechyńska-Hebda and Karpiński, 2013). In a wide range of plant species such a situation leads to an increase in the total pool size of xanthophyll cycle pigments (VAZ pool) and a reduced epoxidation state of these pigments (Demmig-Adams and Adams, 1992). Regulation of the VAZ pool size was shown to be under control of plastid signals (Kawabata and Takeda, 2014). Among the signals originating from high-light-exposed chloroplasts are redox changes and ROS, and metabolites or hormones synthesized in plastids (Bouvier *et al.*, 2008; Dietz, 2015). In comparison with wild-type plants, RNAi-W1 plants have an increased size of the VAZ pool and a reduced epoxidation state of these pigments (Fig. 3). Therefore, they seemingly experience a higher excess of energy in chloroplasts in accordance with the reduced photosynthetic capacity of the plants and a high basal fluorescence (Supplementary Fig. S3). The results show that RNAi-W1 plants are obviously capable of reacting to the EEE situation by increasing the pool of xanthophyll cycle pigments (VAZ pool) and by converting violaxanthin to zeaxanthin. In contrast, the RNAi-W1 transgenic plants are not able to respond to high irradiance by promoting senescence processes. This suggests that WHIRLY1 plays a role in retrograde signalling pathways that are not relevant for the regulation of the VAZ pool. By employing specific inhibitors of the electron transport chain, Kawabata and Takeda (2014) demonstrated that in a photoautotrophic cell culture of *A. thaliana* the VAZ pool size and the transcription of genes involved in xanthophyll biosynthesis such as ß-carotene hydroxylase genes are at least in part controlled by the redox state of the plastoquinone pool. In contrast, the expression of those genes was not affected by the ROS hydrogen peroxide (Maruta *et al.*, 2012) or singlet oxygen (Ramel *et al.*, 2013).

Originally, WHIRLY1 was considered as a nuclear transcription factor affecting senescence by binding to the promoters of specific genes such as *HvS40* (Fig. 4) (Krupinska *et al.*, 2014*a*). In order to investigate whether senescence-associated changes in gene expression are affected by WHIRLY1, Nanostring analyses were performed for 27 genes using RNA from leaves of the wild type and of the RNAi-W1-7 line collected during development at high irradiance. Of particular interest was the expression of genes containing ERE-like elements in their promoters, namely *HvS40*, *WRKY12*, *PR1*, and *PR10* (Fig. 4). The expression data indicate that all of these genes have higher transcript levels before the onset of senescence and hence might be repressed by WHIRLY1. Besides the predicted target genes of WHIRLY1, *CAT2* and *GS1* genes are also repressed by WHIRLY1 by factors of 10 and 4, respectively, at 10 das (Fig. 5; Supplementary Fig. S2). *CAT2* belongs, like *ptOX* and *Cu/ZnSOD1*, to those genes controlling ROS levels. In the RNAi-W1 plants, they also show enhanced expression (Fig. 5; Supplementary Fig. S2) and the Cu/ZnSOD1 protein amount is elevated throughout leaf development (Fig. 6). The enhanced expression of the genes involved in the control of ROS levels is in accordance with a higher excess energy situation in the RNAi-W1 plants compared with wild-type plants.

Abiotic stress factors such as drought and high light are known to reduce photosynthesis and, as a consequence of overexcitation of the photosynthetic apparatus, ROS accumulate, leading to premature senescence (Pintó-Marijuan and Munné-Bosch, 2014). Indeed, thylakoids isolated from senescent leaves were shown to produce more ROS than thylakoids from non-senescent leaves (Krieger-Liszkay *et al*., 2015). EPR measurements revealed that thylakoids from RNAi-W1 plants exposed to high light produce more superoxide/hydrogen peroxide than those from wild-type chloroplasts (unpublished results). This could also cause the reduced assimilation rate of young leaves of RNAi-W1 plants compared with the wild type (Fig. 2D). However, despite the higher excess energy situation and enhanced ROS production, senescence of the RNAi plants is slowed down in high light (Figs 1–3).

Besides ROS, hormones and sugars are known to promote senescence. In particular, chloroplast-derived hormones are well-known regulators of leaf senescence (Buchanan-Wollaston *et al.*, 2005; Gregersen *et al.*, 2014). The levels of ABA (Samet and Sinclair, 1980), salicylic acid (Morris *et al.*, 2000), and jasmonic acid (He *et al.*, 2002) were shown to increase in senescing leaves. Stay-green plants are expected to have lower levels of these hormones. An analysis of hormone amounts in leaves of the RNAi-W1 and wild-type plants revealed, however, that the levels of these hormones are not reduced in leaves of the RNAi-W1 plants but rather they are enhanced (Supplementary Fig. S4). Sugars are known to be strong repressors of photosynthesis-related gene expression, and their accumulation can lead to premature senescence (Rolland and Sheen, 2005). Sugars have also been suggested to play a role in the co-ordination of chloroplast and nuclear gene expression during acclimation of plants to high light (Häusler *et al.*, 2014). Determinations of glucose, fructose, and sucrose in leaves from RNAi-W1 and wild-type plants grown under high light did not show significant differences (Supplementary Fig. S5).

The measurements of hormones (Supplementary Fig. S4) and sugars (Supplementary Fig. S5), and changes in expression of photosynthesis and senescence associated genes (Fig. 5; Supplementary Fig. S2) and in the abundance of photosynthesis proteins (Fig. 6) indicate that the typical senescence-associated features are not impaired in the RNAi-W1 plants. With the exception of darkness (Fig. 7), it seems rather that the diverse plastid signals known to induce senescence processes are not properly sensed in the RNAi-W1 plants. This suggests that WHIRLY1 is not involved in a specific plastid signalling pathway, but rather in a master mechanism acting downstream of multiple plastid signals and integrating diverse plastid signals (Richly *et al.*, 2003).

Considering that the abundance of WHIRLY1 is higher in non-senescent leaves, it is likely that the protein action resulting in enhanced reactivity towards high light already occurs in non-senescent leaves. Whether this activity of WHIRLY1 is associated with the nucleus or with chloroplasts remains, however, to be investigated. Recently, it has been demonstrated that WHIRLY1 is required for senescence-associated changes in histone modifications occurring at the *HvS40* gene (Janack *et al.*, 2016). It is hence possible that changes of the epigenetic landscape are required for senescence promotion in response to stress. Thereby the abundance of WHIRLY1 might depend on stress-induced changes in chloroplasts as proposed by Foyer *et al.* (2014). In addition, or alternatively, the lack of senescence promotion by light might be related to direct actions of WHIRLY1 inside chloroplasts. It is known that leaves of RNAi-W1 plants have higher levels of plastid DNA and disorganized nucleoids (Krupinska *et al.*, 2014*b*). Nucleoids are closely attached to thylakoids and can have effects on the structure and function of the photosynthetic apparatus. A reduced compactness of nucleoids and loosening of the contact with thylakoid membranes could be responsible for the reduced PSII efficiency and reduced photosynthetic carbon fixation of RNAi-W1 plants. It is also possible that the proper organization of nucleoids is required for WHIRLY1-independent plastid signalling in response to high light. Indeed nucleoids were found to contain several proteins involved in plastid to nucleus signalling (Melonek *et al.*, 2016). Their effectiveness in plastid signalling might be determined by WHIRLY1-dependent changes in the architecture of nucleoids (Melonek *et al.*, 2016).

In conclusion, the slowing down of light-dependent senescence processes but not of dark-induced senescence in WHIRLY1-deficient barley plants indicates that WHIRLY1 is involved in sensing and signalling of light intensity. Whether WHIRLY1 plays a direct role in perception of light intensity or indirectly affects other processes involved in sensing of light and in the plastid to nucleus signalling remains to be investigated.

## Supplementary Material

Supplementary Data
