# Supplementary material for: Acceleration of leaf senescence is slowed down in transgenic barley plants deficient in the DNA/RNA-binding protein WHIRLY1
Source: J Exp Bot. 2017 Jan 28;68(5):983–96. doi: 10.1093/jxb/erw501 (PMC5441857; doi:10.1093/jxb/erw501)
Supplement: Supplementary Data [file erw501_Supplementary_Data.zip › supplementary_figures_S1_S5_tables_S1_S2.pdf]

TITLE PAGE

Acceleration of leaf senescence by light is slowed down in transgenic barley plants deficient in the DNA/RNA binding protein WHIRLY1

Weronika Kucharewicz[1], Assaf Distelfeld[2], Wolfgang Bilger[1], Maren Müller[3], Sergi Munné-Bosch[3], Götz Hensel[4], Karin Krupinska[1,*]

[1] Institute of Botany, Christian-Albrechts-University of Kiel, Kiel, Germany
[2] Department of Molecular Biology and Ecology of Plants, University of Tel Aviv, Israel
[3] Department of Evolutionary Biology, Ecology and Environmental Sciences, University of Barcelona, Spain
[4] Plant Reproductive Biology, Leibniz Institute of Plant Genetics and Crop Plant Research (IPK), Seeland/OT Gatersleben, Germany

***** Author to whom correspondence should be addressed: Karin Krupinska

E-Mail: kkrupinska@bot.uni-kiel.de

Tel.: +49-431-8804240; Fax: +49-431-8804238

Figure S1.

Three independent transgenic lines with RNAi mediated knockdown of the *WHIRLY1* gene (W1-1, W1-7, W1-9) are compared to wild-type plants. Plants were grown adjacent to each other in a glass house.

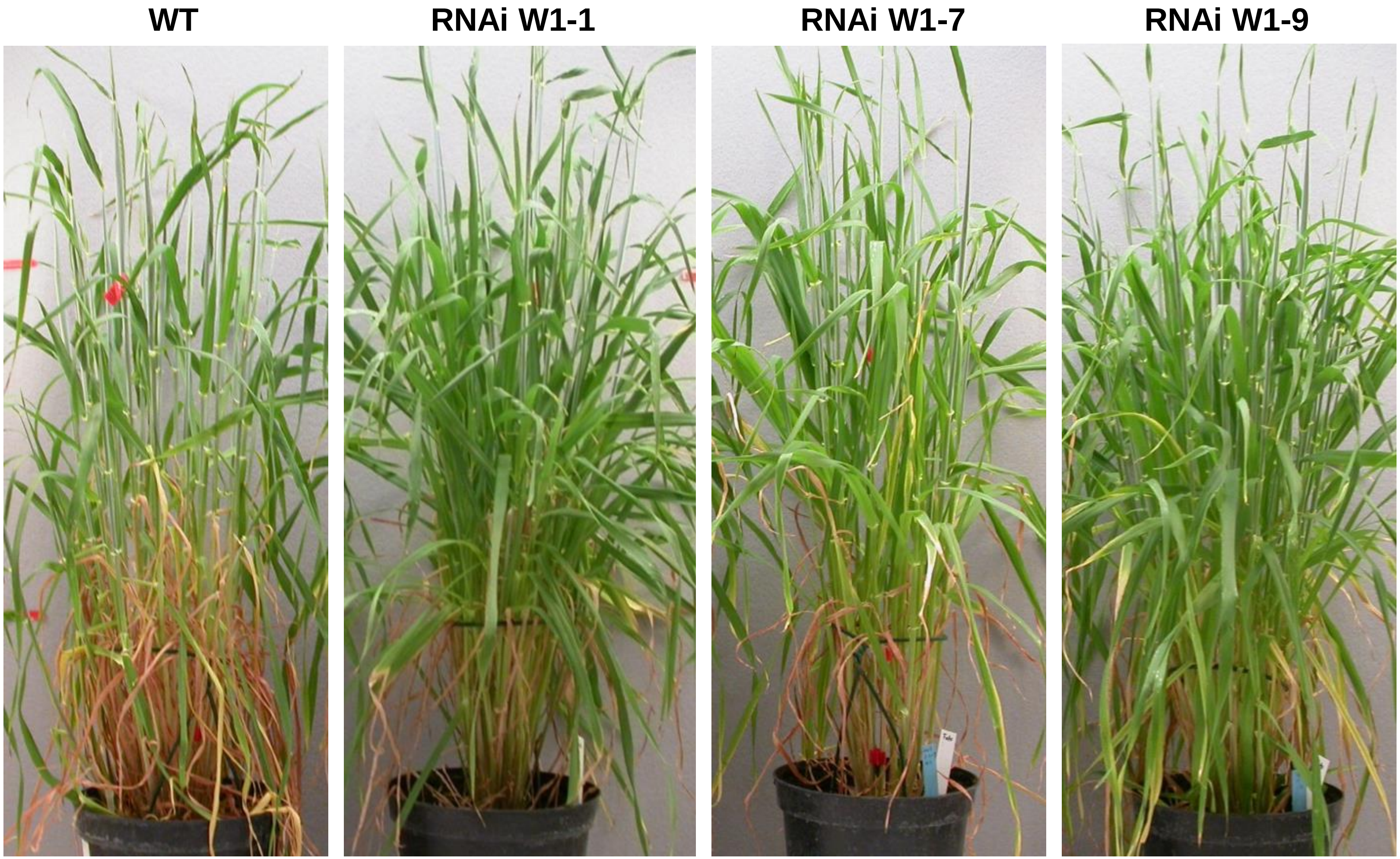

Figure S2.

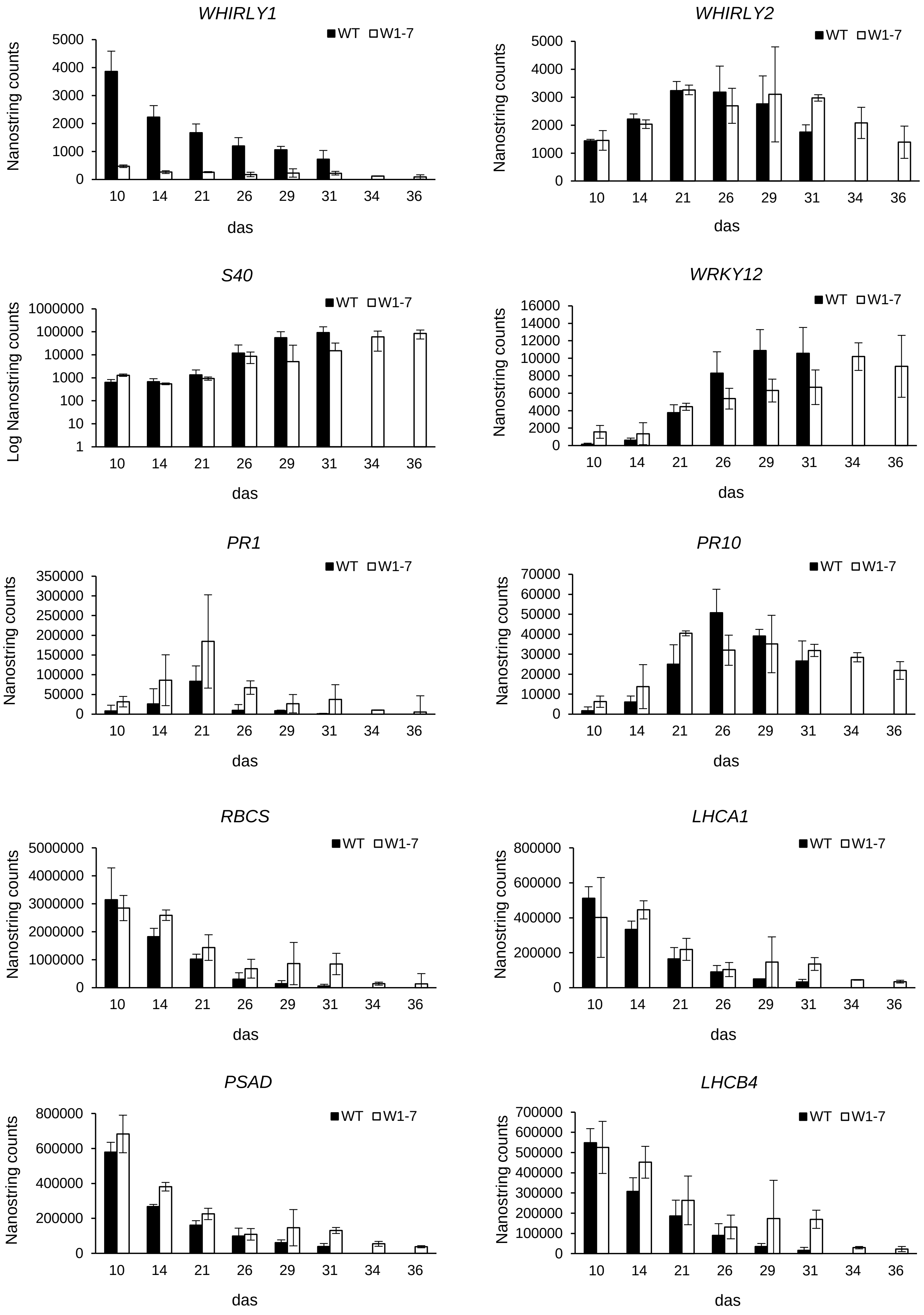
WHIRLY1
WHIRLY2
S40
WRKY12
PR1
PR10
RBCS
LHCA1
PSAD
LHCB4
WT
W1-7
Nanostring counts
Log Nanostring counts
das
10
14
21
26
29
31
34
36

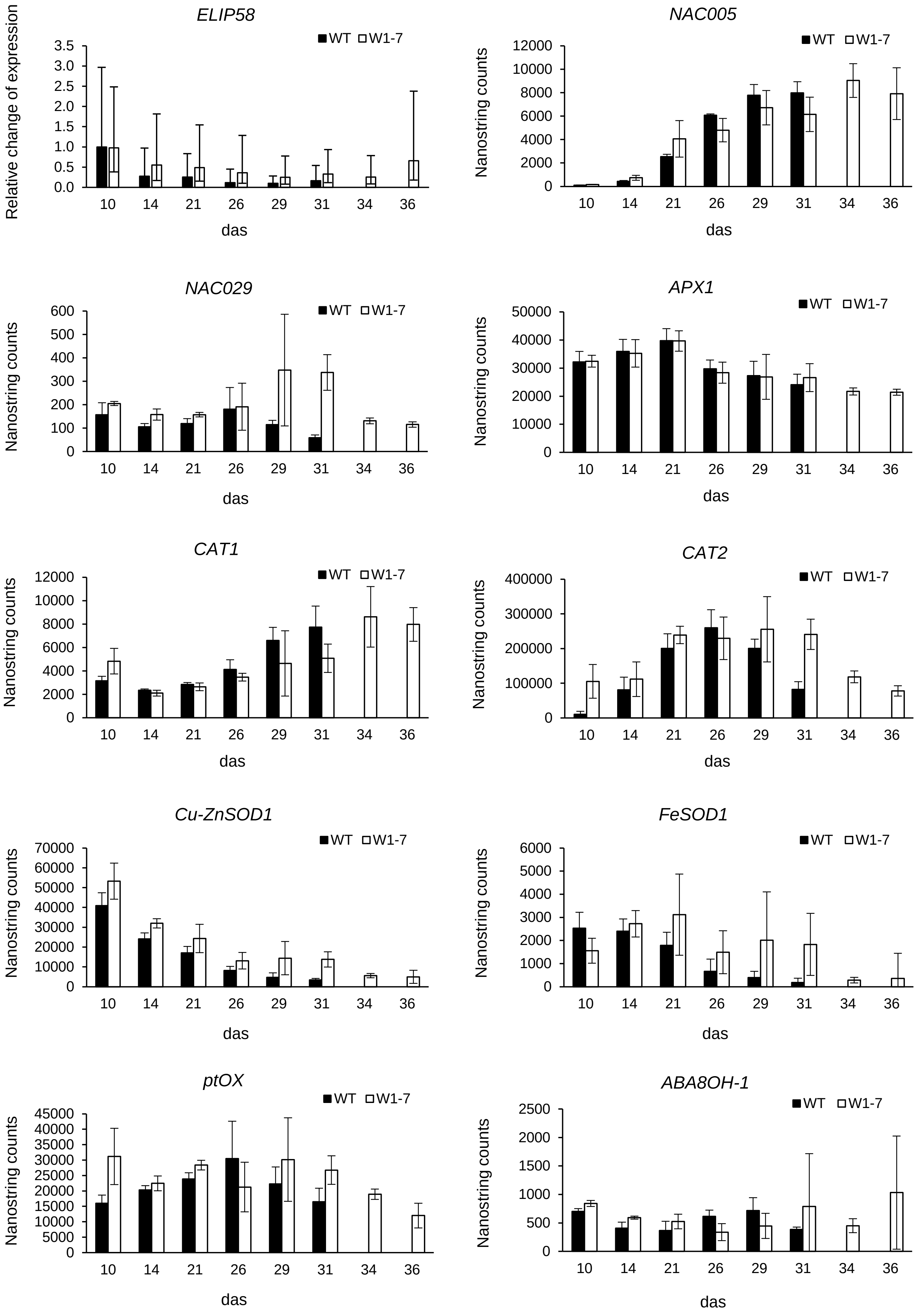
ELIP58
Relative change of expression
Nanostring counts
NAC005
NAC029
APX1
CAT1
CAT2
Cu-ZnSOD1
FeSOD1
ptOX
ABA8OH-1
WT
W1-7
10
14
21
26
29
31
34
36
das

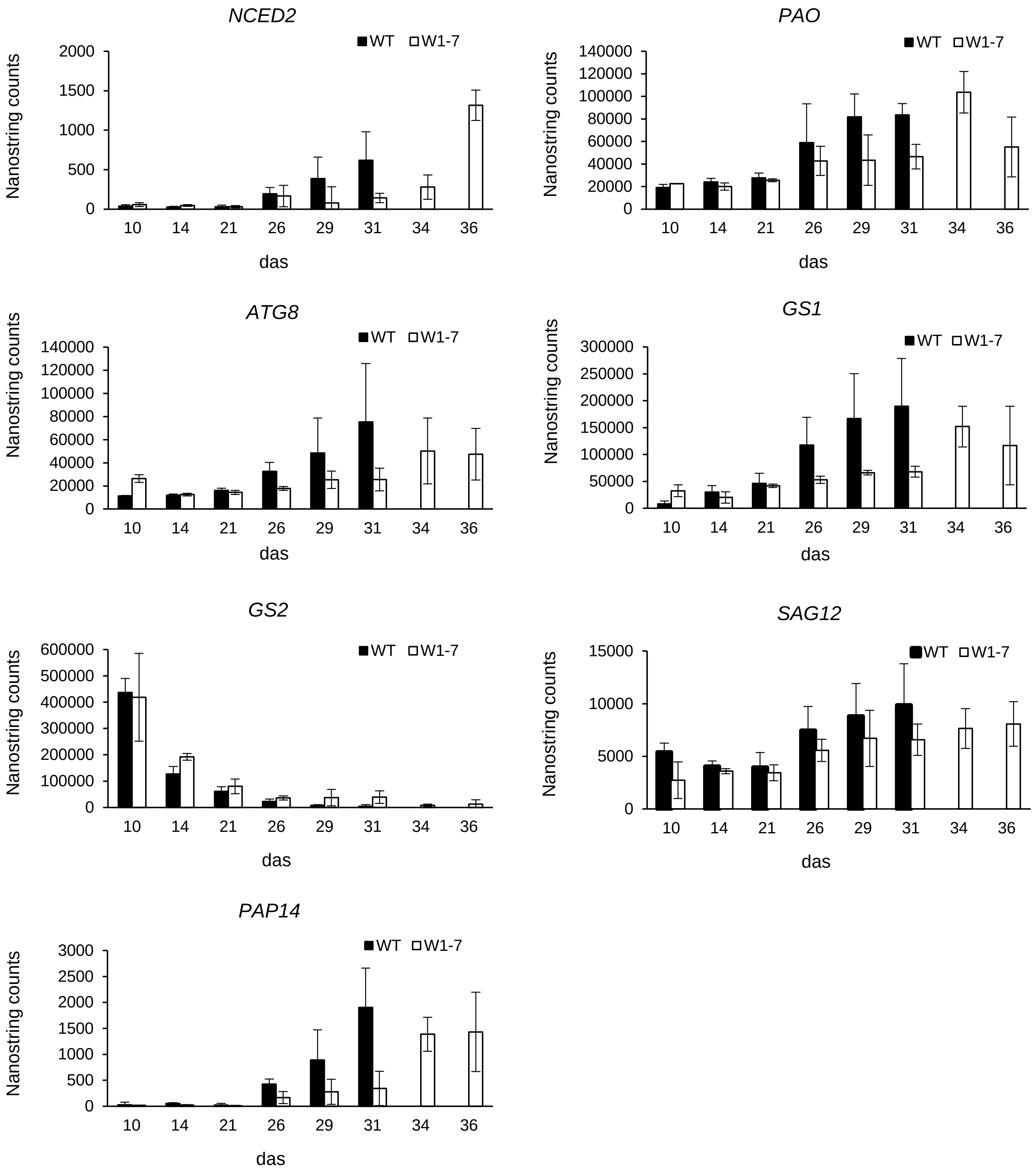

Nanostring gene expression analysis. Relative mRNA levels were analysed during development of primary foliage leaves from wild type and RNAi-W1-7 plants grown at high irradiance. Samples representing 6 (wild type) or 8 stages of development (RNAi-W1) as described in Figure 5. The mean of raw counts and standard deviations from three biological replicates are shown.

Figure S3.

In vivo Chl a fluorescence was measured using the Dual-PAM-100 (Walz, Effeltrich, Germany; Pesaresi *et al.,* 2009) on wild type and RNAi-W1-7 leaves at different time points during their development showed as percentage of relative chlorophyll content (SPAD) of the WT leaves.

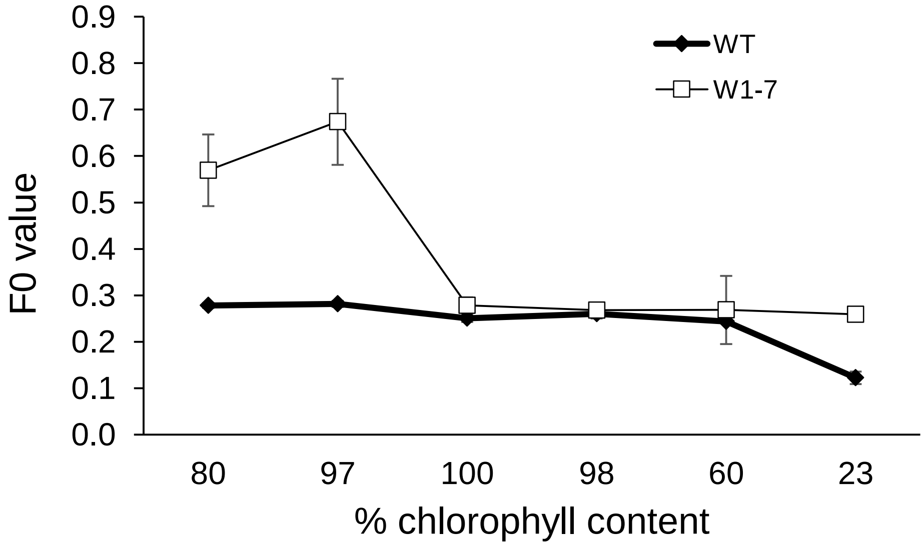

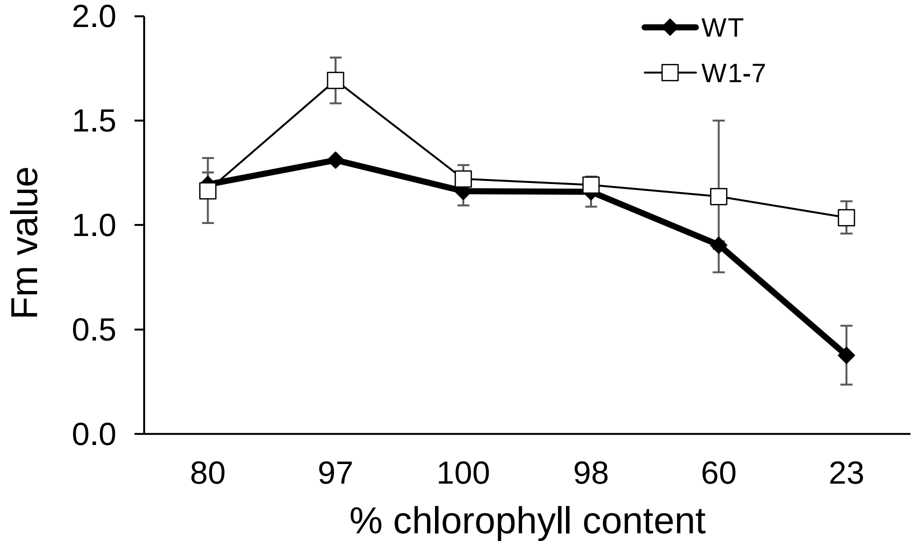

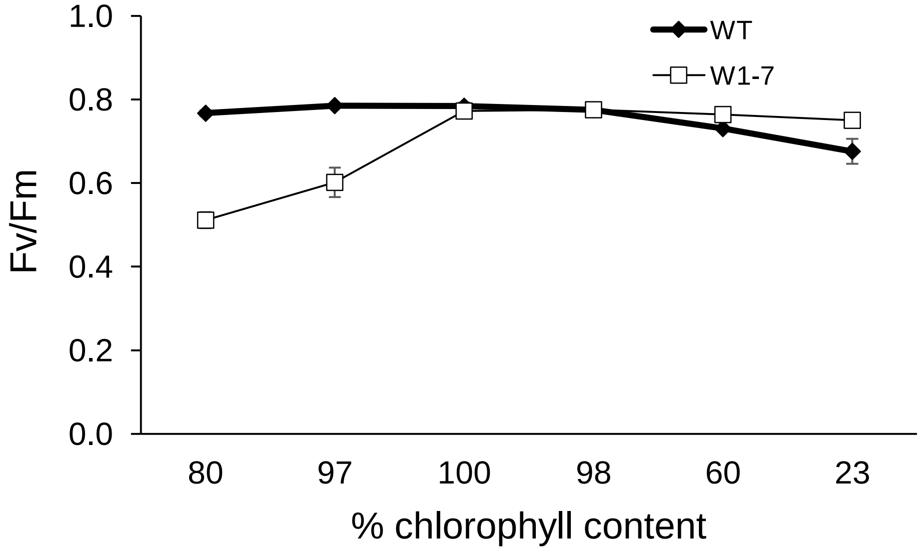

Figure S4.

Hormones and relative chlorophyll content of the primary leaves of wild-type and RNAi-W1-7 plants. Plants were grown at ~150 µmol s^{-1} m^{-2} until 10 das and then transferred to 500 µmol m^{-2} s^{-1}. Mean (+/-SD values) are based on the values from three replicates. Endogenous concentrations of abscisic acid (ABA), salicylic acid (SA), jasmonic acid (JA), auxin (IAA) and cytokinin (t-Z) were extracted and quantified from 100 mg of tissue as described by Müller and Munné-Bosch (2011).

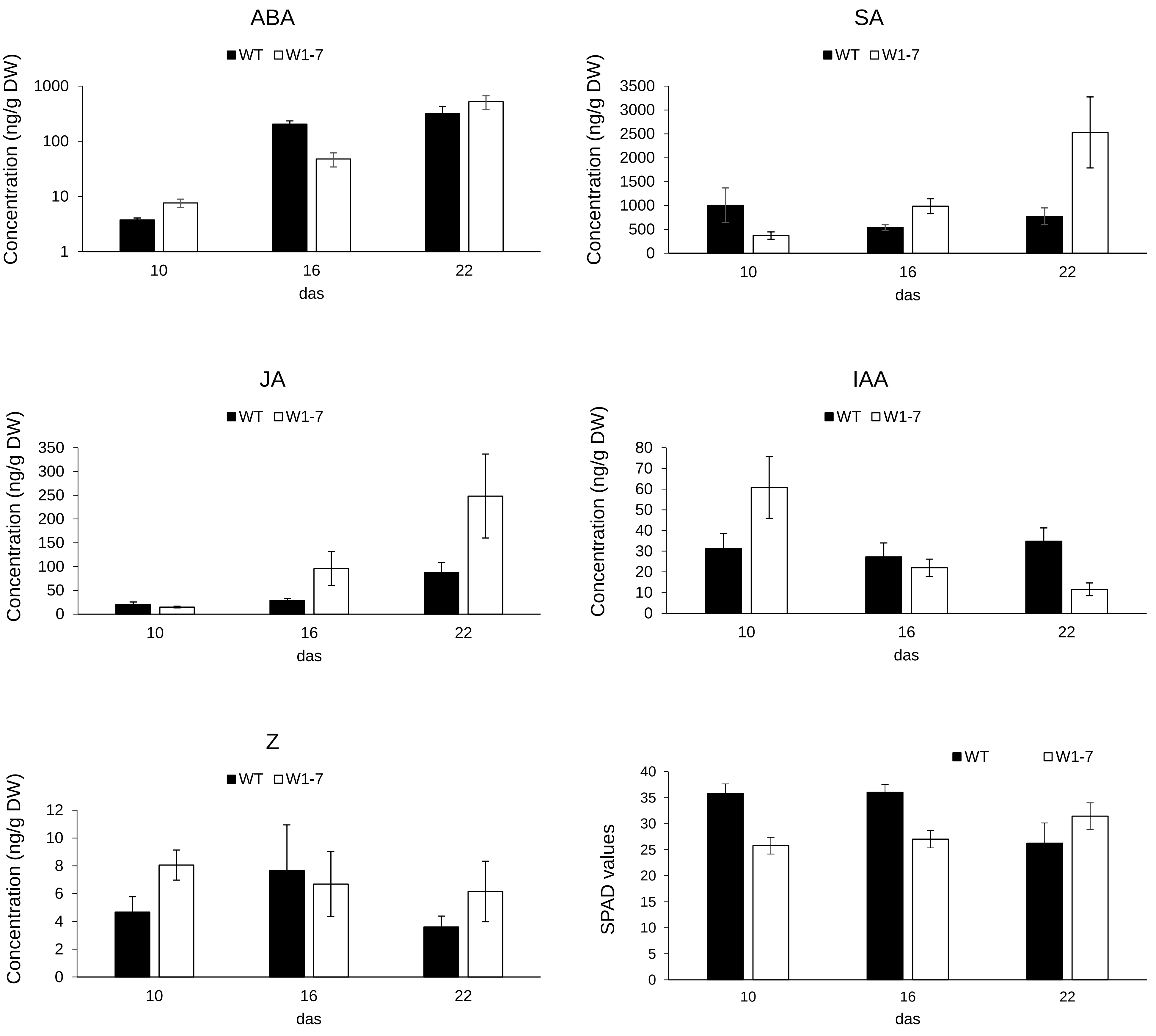

Figure S5.

Sugar contents of the primary leaves of WT and W1-7 plants grown at 350 μmol photons m^{-2} s^{-1} at different days after sawing. Glucose, fructose and sucrose amounts in the samples were determined using enzymatic assay (CAT# 10-716-260-035 R-Biopharm, Darmstadt, Germany). Mean (+/-SD values) are based on the values from three replicates.

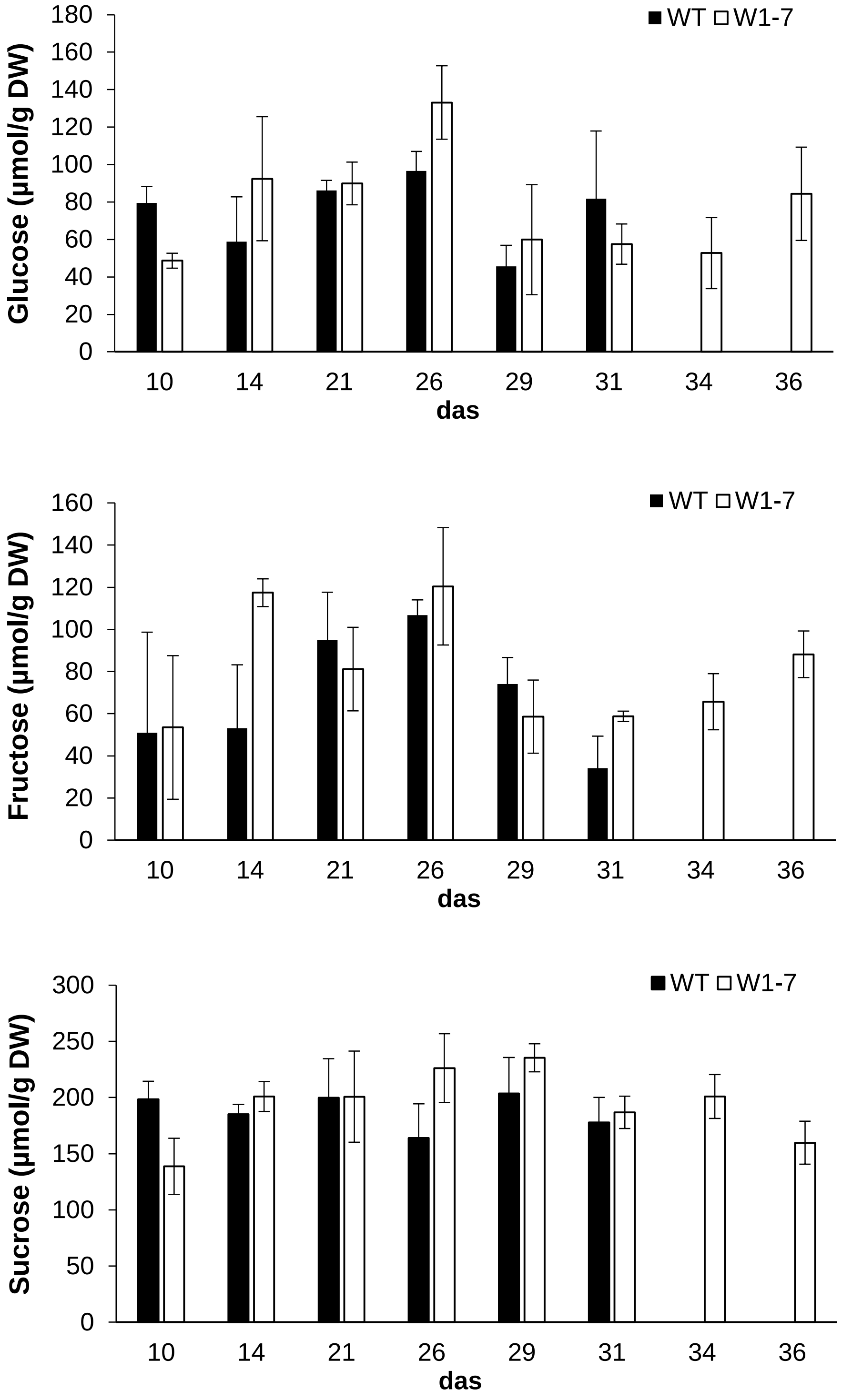

Table S1. A list of genes and sequences used for Nanostring expression analysis.

Gene name	Accession Number	Target region	Target sequence
WHIRLY1	AK365452.1	491-590	GTTCTCATTATCTGTGTGGGAGATGGGAACCTTGCTTACTCTTGGTCTAACAGATTC GTGCGAGTTCTTTCATGATCCTTTCAAGGGAAGGAGCGACGAA
WHIRLY2	AK249976.1	81-180	GTCACTGGCCTCAAAGATGCTCTCTGGAGCGGTTCATTGACATTCAAGCATGCACT TTCGACTTCAGCTGCAAATGTTGATGAGAATGCATCTGCTAAGA
NAC005	AK251058.1	106-205	CCAAGCTCTAGCTAGCCAGCTAGGGTTTTCGTCCTCGAGCGTTGCGTGGCGTGGC GTGGCGATGATCATGTCCGACCCGGCCATGCTGCCGCCGGGCTTC
NAC029	EU908210.1	1232-1331	GCGGCGAGGGCCACCAAACAGTTCCTCGCCCCGTCGTCCTCAACCCCGTTCAACT GGCTCGAGGCGTCAACCGGTGGCAGCATCCTCCCACAGGCAAGGA
WRKY12	AK354853.1	606-705	ACCCAATTGAGAAGTCTAATGACAACTTTGAGCACATACTCACCCAGATGCAGGTCT ATTCAGGCCTCAACAATGTCTCCCAAACCTTTGGCAACCAACA
MYB	Bar_MYB.1	7-106	GAGGCGCAGGAGGCCTGGGAGCACTACCAGGCGCTCGTGGCCGACGTCGACCTC ATCGAGCGCGGCGCGGTGGACACGCCCGACTGCTGGGACGATGACG
RbcS	U43493.1	221-320	ACTCAGCACGGAGGCCCTCCTTAAGCAGGTCGACTACCTGATCCGCTCCAAGTGG GTGCCTTGCCTCGAGTTCAGCAAGGTTGGGTTCATCTTCCGTGAG
LHCA1	AF218305.1	1-100	GGCCGCGCAGCAAGGAGCAGCCAGCATCGAGAGAGCAGCAATGGCTATGGCGTC GTCAAGCGGGCTCAGGAGCTGCAGCGCCGTGGGCGTGCCGAGCCTG
PsaD	M98254.1	631-730	ACCGGCAAGAACTCCTTCGACATCTAATCTCGCTCGTACGTCGTATATGTGCATGCT TATGCGTACGTGCTAGTTCATCGACCGGTAGTTATTGGCGGGT
LHCB4	AJ006296.1	176-275	CCACCTCAGCGACCCGCTGCACACCACCATCTTCGACACCTTCGGCTCCTCTTAAG TGGTCGGTGGTCGAGCAGTTTCTGTCAGCTCAGCTGCAAAATGG
APX1	AJ006358.1	596-695	TTTGAGGGACCCTGGACAAGGAACCCTTTGAAGTTTGACAACTCTTACTTCACGGA GCTTTTGAGTGGTGACAAAGAGGGACTTCTTCAGCTTCCAAGTG
CAT1	U20777.1	171-270	TCACCGTCGGACACCGAGGACCTATCCTCCTTGAGGATTACCATCTGATTGAAAAG CTTGCACAATTTGACCGGGAACGCATACCTGAACGTGTTGTTCA
CAT2	U20778.1	21-120	CCCAGCTTAGCTACGCTACTTCACTCACTCGAGGAAGGATGGATCCCTGCAAGTTC CGGCCGTCGAGCAGCTTCGACACCAAGACGACGACGACGAACGC
Cu-ZnSOD1	HM537232.1	296-395	TTCGTATCACTGGACTTACTCCTGGACTTCATGGTTTCCACCTCCATGAGTTCGGTG ACACGACTAATGGATGCATATCAACAGGGCCACATTTTAACCC
FeSOD1	AK375983.1	526-625	GGATTTCGGCTCCTTTGTTAATTTCAGGGAGGAGTTCATGCGCTCGGCGCTGTCGT TGCTGGGGTCTGGTTGGGTTTGGCTTGTCTTGAAGAGAAGCGAG
ptOX	AK359478.1	436-535	CGAGCAGTCCTTCAACGTATTCCTCACGGACACCGTCATCCTGATACTCGATATTCT GTACCGCGACCGCGACTACGCCAGGTTCTTCGTGCTCGAGACC
PR1	Z21494.1	473-572	CCGCGGCGTCTTCATCACCTGCAACTACGAGCCCCGCGGGAATATCATTGGACAG AAACCATACTAACCGTGATGCACGTCATCGTCTCTCGTCCCTAAT
PR10	AY220734.1	1-100	GAATTCGGCACGAGCAGCAGCGCTAGTGTGCAGCGAGCCAGTGACCGACGGACAA GAACAAGATGGTCGCCGGCTGTGTCATCACCGAGCAGTGTGCCCT
S40	AK248955.1	401-500	ATATCAGGAACCTCGTCCTCAGGATGACCGGATTCATCGAGAAGTGAAATGGATTC TACACCGTGGATATATTGCTTCGTTGCTTGGGAACGTCCATGGA
ABA8OH-1	DQ145932.1	1-100	GTTGCAGGTTGCAGGTAACAGAACCGAAGAAATCCTTTTGGAATGGGTGCCTTCAT CCTCCTCCTCTGCTTGCTCGTGCCGTTGGTGCTCGTGTGCGCCG
NCED2	DQ145931.1	720-819	CCAACGCCGGCCTGGTCTACTTCAACGGCCACCTCCTCGCCATGTCCGAGGACGA CATCCCGTACCACGTCCGCGTCACCGACGACGGCGACCTCCAGAC
ATG8	AK251678.1	166-265	GCTAACCGCATAAGAGAGAAGTACTCTGACAGAATTCCTGTGATCGTTGAGAAGGC TGGGAAGAGTGATATTCCTGACATTGACAAGAAAAAGTACCTTG
GS1	KF815945.1	1191-1290	GTTGCATACTACTCCTAGTCCGCTTAGGTAGGTCACATCATCATGGTCATCTCATCA GGGTGTCTGGTCTCTCTTCTCGTTCTCGTCTTTGGGTGGGTGG
GS2	AK360336.1	736-835	ATACTACTGCGCCGTAGGATCAGACAAGTCATTTGGCCGTGACATATCAGATGCTC ACTACAAGGCGTGCCTTTACGCTGGAATTGAAATCAGTGGAACA
SAG12	AM941123.1	496-595	AGAGGCGCCGTCACGGCGGTGAAGAACCAAGGCCAGTGCGGATCGTGTTGGGCA TTCTCCACCGTAGCGGTGATTGAAGGAATCCACCAAATCAAGACCG
PAP14	AM941124.1	525-624	TCTCGACGATCGCGGCCGTGGAGGGCATCAACGCTATCCGCTCTAAGAACCTCAC GTCTCTGTCCGAGCAGCAGCTCGTGGACTGCGACACGAAGTCCAA
PAO	AK365037.1	1141-1240	GCAGCTTGTCCCTCGATGGTACGAACACTGGACCTCGAATTTGGTCTACGATGGTG ATATGATCGTGCTTCAAGGCCAAGAGAAGGTTTTCCTGTCTGCA
Act	AK248710.1	341-440	AGGCCAACAGAGAAAAAATGACCCAGATCATGTTTGAAACCTTCAGTTGCCCAGCA ATGTATGTTGCAATCCAGGCCGTTCTGTCCTTGTATGCCAGCGG
CDC	AK376851	1906-2005	ATCATAGACCCTGCCCTGCTTAGGCCAGGGCGTCTTGATCAGCTTATCTACATCCC TCTTCCTGATGTCGATTCGAGGCACCAGATCTTCAAAGCTTGCC
RLI	AK355298	1194-1293	GCCAGATACCATGGAAGGAACCGAGGTTGAAATTCCTGAATTCAATGTGTCCTACA AGCCTCAAAAGATCAGTCCAAAATTTCAGCATCCAGTGAGGCAC

Table S2. Sequences of the primers used in qPCR analysis.

Gene name	Accession Number	Primer sequences
HvActin	AK356840.1	F: 5’-GGAAATGGCTGACGGTGAGGAC-3’<br>R: 5’-GGCGACCAACTATGCTAGGGAAAAC-3’
HvSP2	AK249101	F: 5’-GAAGGAGTATGCTGGCAAAA-3’<br>R: 5’-TGACCTTGAATGGGAAGAAA-3’
HvELIP58	X15693.1	F: 5’-GACTCAGCCAAGGAGACGAC-3’<br>R: 5’-GGTTAGCCTTCTTGGTCACG-3’
